# Utilization of Cassava Starch–Glycerol Gel as a Sustainable Material to Decrease Metal Ion Surface Contamination

**DOI:** 10.3390/gels11050363

**Published:** 2025-05-14

**Authors:** Rezky Anggakusuma, Gemilang Lara Utama, Dadan Sumiarsa, Permata Apriliani Dewi Muslimah, Ali Asgar

**Affiliations:** 1 Doctoral Program on Environmental Sciences, Graduate School, Universitas Padjadjaran, Jl. Dipati Ukur No. 35, Bandung 40132, Indonesia; 2 Directorate of Laboratory Management, Research Facilities, and Science and Technology Park, BRIN, Jl. Sangkuriang No. 1–5, Bandung 40135, Indonesia; 3 Center for Environment and Sustainability Science, Universitas Padjadjaran, Jalan Sekeloa Selatan 1 No. 1, Bandung 40132, Indonesia; 4 Department of Chemistry, Faculty of Mathematics and Natural Sciences, Universitas Padjadjaran, Jatinangor, Sumedang 45363, Indonesia; dadan.sumiarsa@unpad.ac.id; 5 Chemistry Degree Program, Faculty of Science and Technology, Muhammadiyah University of Sukabumi, Kota Sukabumi 43113, Indonesia; permata011@ummi.ac.id; 6 Research Center For Agroindustry—BRIN, KST Soekarno Cibinong, Jl. Raya Jakarta-Bogor KM 46, Cibinong, Bogor 16911, Indonesia; alia005@brin.go.id

**Keywords:** cassava starch, decontamination gel, sustainable material, metal ion, contamination

## Abstract

Many studies have examined the ability of polymer-based gels or hydrogels to serve various purposes, particularly as absorbents. Several studies have reported that polyvinyl alcohol (PVA), with specific compositions and additives, is an absorbent and a decontamination material usable for heavy metals and radioactive substances. PVA has a high cost and is slowly degradable under anaerobic conditions. This study investigated the potential of natural materials, namely cassava starch, which is an environmentally friendly, non-toxic, and readily available gel-forming polymer that, notably, is inexpensive in Indonesia. The FTIR analysis showed a bond and polymer formation between cassava starch and glycerol. The cassava starch–glycerol–water mixture was applied to media such as glass, aluminum plates, and ceramics contaminated with heavy-metal stable ions which correspond to a radionuclide. The media, stored at room temperature for 24 h, becomes a film. According to the SEM and XRF results, the gel becomes a film that binds and absorbs metals when dried. The SEM results showed the presence of metals corresponding with the sources of contamination, and the XRF results showed that the quantity of metals absorbed was large. The cassava starch gel absorption results indicated the formation of an amorphous compound, as indicated by the XRF results. Based on all the analyses, the cassava starch–glycerol gel has enormous potential. It is almost equivalent to a PVA gel as an absorbent material and heavy-metal decontamination material, when used for radioactive decontamination on the material’s surface.

## 1. Introduction

Indonesia has three research reactors: the TRIGA 2000 Reactor Bandung (operating since 1965), the Kartini Reactor Yogyakarta (operating since 1979), and the GA Siwabessy Multipurpose Reactor (operating since 1987) [1,2,3]. The oldest is over 50 years old and has an expiring operating permit. Under applicable regulations, the TRIGA 2000 Bandung reactor must prepare an appropriate decommissioning program consistent with provisions from the Indonesian Nuclear Energy Regulatory Agency (BAPETEN) and the International Atomic Energy Agency (IAEA) [1,2,3,4].

Nuclear-plant decommissioning refers to the activities carried out after a nuclear reactor’s operating duration or use permit has expired, or when it will no longer be used permanently. Decommissioning is a complex and drawn-out procedure because of several factors, including safety, health, security, and socioeconomic factors. Decommissioning nuclear plants involves removing the fuel from the reactor core, decontaminating the facility, and destroying the reactor facility; the decommissioning program must determine the radioactivity, radiation dose rate, and contamination level associated with this process [5,6,7,8,9,10,11,12].

To comply with the radiation protection program, the cutting method must be determined, the position or cutting area simulated before disassembly, and the likelihood of contamination by radionuclides or activating materials must be determined. Before and after disassembly, decontamination of the surfaces of the reactor facility or the surrounding area must be performed. The decontamination techniques include mechanical, chemical, electrochemical, and washing-based methods [13]. The chemical decontamination includes chemical solutions, foam, gels, and multiphase treatments; the mechanical decontamination provides for water flushing, vacuuming, wiping, scrubbing, blasting, steam cleaning, high- and ultrahigh-pressure water jetting, grinding, milling, and sprouting; the emerging technologies include light ablation, microwave scabbling, thermal degradation, and electromigration; and the other methods include electropolishing, ultrasonic cleaning, and melting [14,15,16]. A gel can reduce contamination by 10–100%, whereas a foam or jet is associated with a highest decontamination value of 30% [17]. Decontamination can reduce the amount and volume of the solid waste produced by concrete nuclear facilities [18]. The method or type of decontamination affects the overall decommissioning cost. Decontamination costs include materials, consumables, and other expenses, such as labor costs and the processing of decontamination waste [16,19].

Several radionuclides, such as Co-60, Cs-134, Cs-127, Sr-90, U-238, I-129, I-131, Te-129, Ag-110, Th-232, Pu-238, Pu-239, Pu-240, Ir-129, Am241, Tc-97, Tc-98, Tc-99, Zr-93, Zr-95, Fe-55, Nb-94, and other radionuclides that result from fission reaction activation, are contaminants in the research and reactor areas, based on past experiences of decommissioning research reactors. These radionuclides are the results of fission reaction activation. As per the IAEA Technical Report, radionuclides such as H-3, C-14, Na-22, Cl-36, Ar-39, Ca-41, Mn-54, Fe-55, Ni-59, Ni-63, Co-60, Zn-65, Mo-93, Zr-93, Nb-94, Ag-108m, Ag-110m, Sb-125, Ba-133, Cs-134, Eu-152, Eu-154, Eu-155, and Ho-166m are frequently discovered during reactor decommissioning [13,20].

One of the methods of decontamination frequently used involves coating the material’s surface with a gel made from a polymer [16,21,22]. Researched using colloidal gel to coat small contaminated objects. The research results stated that combining carrageenan and silica decontamination gel can reduce the contamination from Co-60 [23]. Moore et al. (2019) performed research on decontamination using a mixture of polyvinylpyrrolidone polymer and found that it could reduce Sr-90 contamination by up to 87% [24]. Research on gels for surface decontamination in nuclear facilities continues to expand, primarily utilizing gel materials composed of organic and inorganic polymer matrices [25,26].

Hydrogel materials for surface decontamination are primarily composed of synthetic or artificial chemical polymers, in particular, polyethylene, polyvinyl, and polyacrylate [24,27,28]. In this study, we prepared a decontamination gel using polymers derived from natural materials, namely, cassava starch flour combined with distilled water and glycerol. The level of cassava starch production on the islands of Java and Sumatra in Indonesia is relatively high, and the country is one of the world’s most significant cassava-exporting countries [29,30]. Cassava starch is environmentally friendly, odorless, and non-toxic. It is a polysaccharide consisting of amylose and amylopectin, and it forms a gel when dissolved in water and heated [31,32,33].

This study used nonradioactive heavy-metal (stable ion) samples to determine the characteristics of cassava starch gel and its ability to bind metals. The results will help us to use starch for radioactive decontamination gels; particularly in Indonesia, which has a reactor planned for decommissioning, this work aims to reduce decommissioning costs and minimize the amount of active waste generated during dismantling.

## 2. Results and Discussion

### 2.1. Starch Cassava–Glycerol Gel

**Figure 1:**
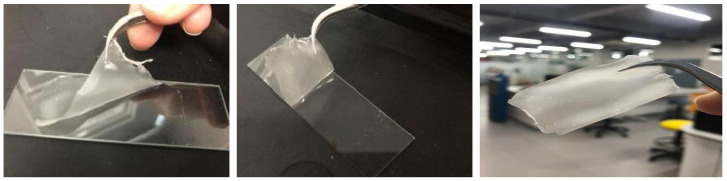
Film gel: starch glycerol (16–10% *w*/*v*).

Figure 1 shows the starch gel films after storage and drying at room temperature for 24 h. In this study, the gel from a cassava starch and glycerol mixture will turn into a transparent white film after 24 h at room temperature.

Starch is an ecologically beneficial natural polymer useful for efforts to reduce pollution or contamination, especially among organic materials, because it has an active O-H group. Chemical or physical processes are used to enhance the functionality of starch as an adsorbent [34]. Starch contains functional groups that can enhance its adsorption performance through various interactions, such as electrostatic interactions or hydrogen bonds [35]. This study utilized starch as a polymer material to absorb or bind metal ions contaminating the surface layers of materials such as glass, aluminum metal sheets, and ceramics. Glycerol improves starch’s ability to interact with metal ions. The combination of starch and glycerol forms a peel-off film. The development of a starch-based peel-off gel formulation could provide an effective solution that is low in cost, easily available using renewable resources, and easily applied in the field, in addition to producing a more sustainable and environmentally friendly waste product.

The starch gel applied to the material surface dries, forming a film that can be peeled off after drying. Five aspects can influence the success of a decontamination gel: the surface absorption of the applied gel, the viscosity of the gel, its ability to bind contaminants, the chemical bonds that comprise the gel, and the potential for interaction with contaminants [36]. This study found that gels with high viscosity are challenging to apply during decontamination and that the drying time for the gel to form a film is more extended. This was the case regardless of whether changes occurred in the film sample, as analyzed using FTIR analysis. Surface absorption is related to the material’s surface area and pore size. This study performed a BET test to determine the area and pore size of the starch–glycerol film.

**Figure 2:**
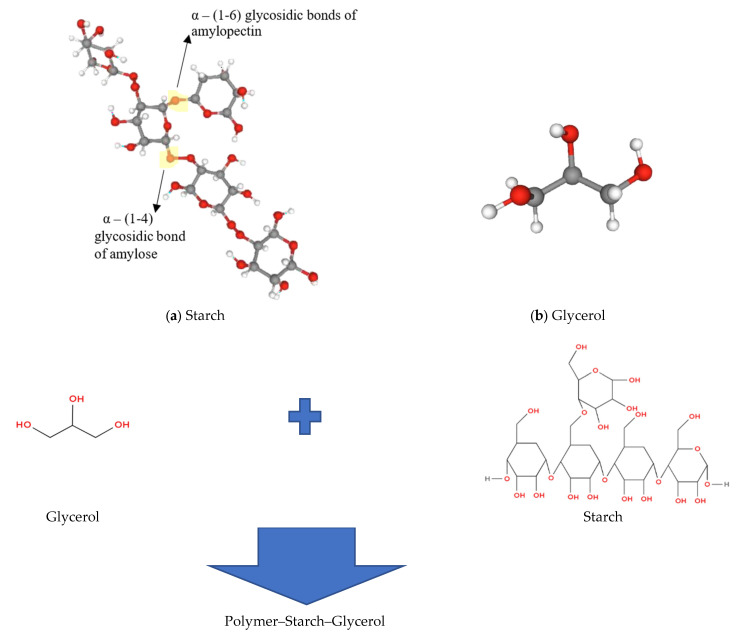

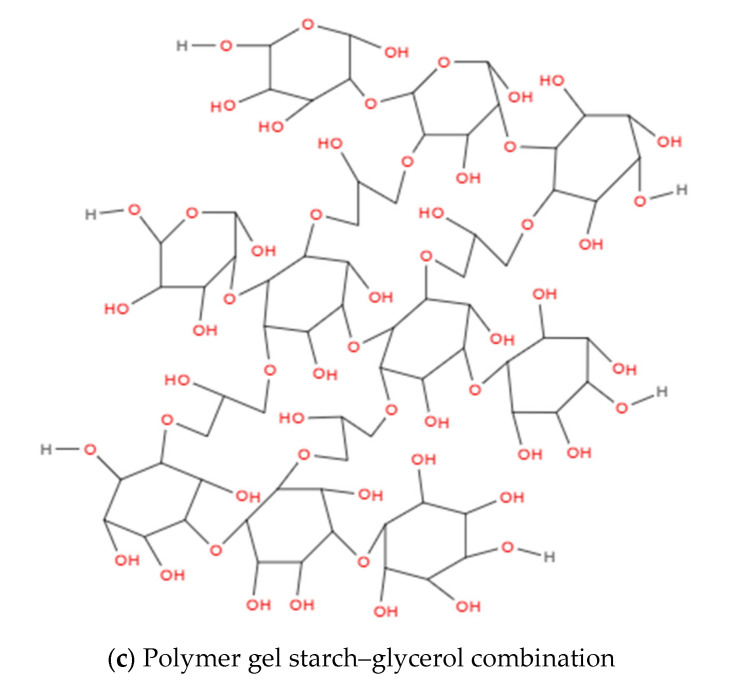
Structural materials and formation of the gel polymer.

Figure 2 shows that Cassava starch consists of two molecules: amylose and amylopectin. Amylopectin has a higher glucose content [20]. Amylose is a linear polymer of α-1,4-linked D-glucose [37]. Glycerol is a basic polyol with a prostereogenic center at position C2 and two primary and one secondary hydroxyl groups. As a result, glycerol can undergo a wide range of chemical changes, including selective oxidation, dehydration, and hydrogenolysis, as well as selective protection and esterification, providing a variety of valuable small-molecule building blocks with high chemical complexity for both polymer and small-molecule chemical synthesis [36].

### 2.2. FTIR Analysis

**Figure 3:**
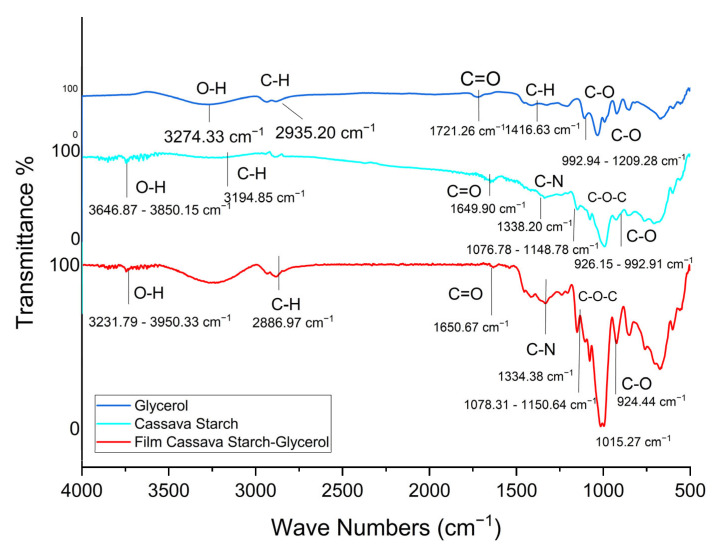
Spectra (IR) from raw material and fixed film.

Figure 3 shows the result of FTIR analysis based on the raw material (glycerol and cassava starch) and the fixed material (film). The results of the glycerol spectrum show the presence of O-H at wavenumber 3274 cm^−1^, C-H at 2935 cm^−1^, C=O at 1721 cm^−1^, C-H at 1416 cm^−1^, and C-O in the range of 992–1209 cm^−1^. These results are based on the FTIR ATR Thermo Nicolet iS5 reference, with a match level of 83.94 % glycerol. The spectrum is also congruent with the results of the other research which details the specific spectrum for glycerol using the following wave numbers: 849 cm^−1^ C-C stretching; 1030 cm^−1^ C-O stretching; 1416 cm^−1^, 908 cm^−1^ C-H2; 1108 cm−1 C-OH; 2880 and 2932 cm^−1^ C-H stretching; and 3286 cm^−1^ O-H stretching [38].

Based on the standard reference, starch cassava is a polysaccharide with a typical FTIR spectrum at wavelengths of 1158, 1080, 1020, 931, and 855 cm^−1^ (800–1200 cm^−1^); which comprises the vibration of the C-OH, CC, and CH side groups and COC glycosidic bond vibrations [39]. In this study, the spectrum of cassava starch has O-H at 3646–3850 cm^−1^, C-H at 3194 cm^−1^, C=O at 1649 cm^−1,^ a range 926–992 cm^−1^, C-N at 1338 cm^−1^, and C-O-C at wavenumber 1076–1148 cm^−1^.

The red line graph in Figure 3 shows the spectrum of the film of cassava starch–glycerol, which includes six types of functional groups O-H, C-H, C=O, C-O-C, C-O, and C-N. The wave number in 3231–3950 cm^−1^ is the O-H group, and 2886 cm^−1^ is the wave of the C-H chain; in the range of 1650 cm^−1^ is the length of the C=O carbonyl group, 1078 cm^−1^ to 1150 cm^−1^ is the C-O-C group of polysaccharides, and 924 cm^−1^ is the C-O carbohydrate group from the glycosidic bond of starch. This follows research conducted by Boonsuk et al. (2020), which states that the glycosidic bonds in cassava starch include the group -OH stretching vibration, the C-O stretching in the C-O-C groups in the glycosidic linkage, and the C-O bending in the C-O-C groups in the anhydrous glucose ring [40].

**Figure 4:**
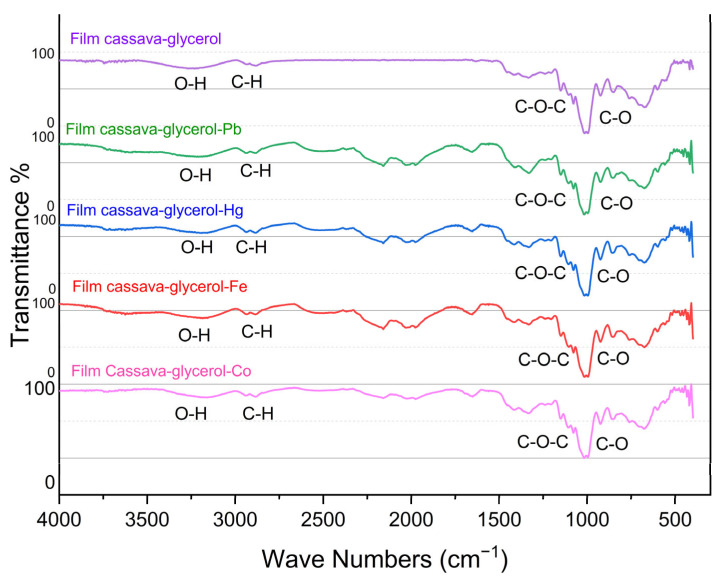
FTIR spectra of metal ions (purple = starch–glycerol film, green = starch–glycerol–Pb (II) film, blue = starch–glycerol–Hg (II), red = starch–glycerol–Fe (II), red light/pink = starch–glycerol–Co (II)).

Figure 4 shows the results of the FTIR test for the starch–glycerol–metal ions, which shows no change in the spectrum of the starch–glycerol film. This may be caused by the low concentration or the small number of metal ions that can affect the group bonds in the starch–glycerol gel.

### 2.3. SEM Analysis

**Figure 5:**
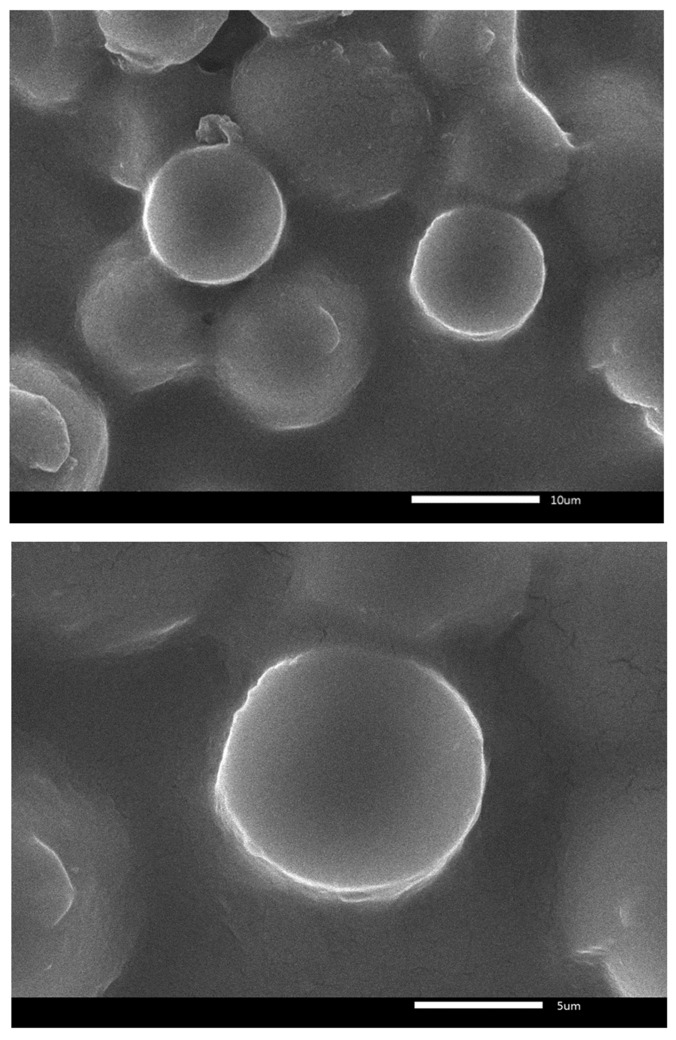
Surface imaging of starch–glycerol at 2500× and 5000×.

**Figure 6:**
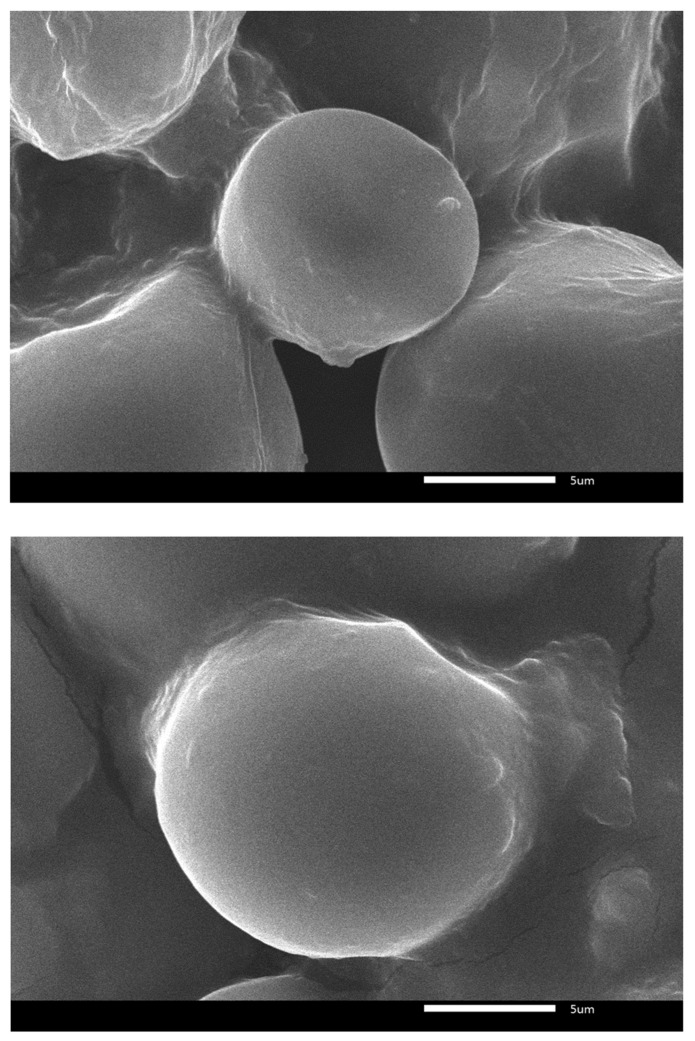

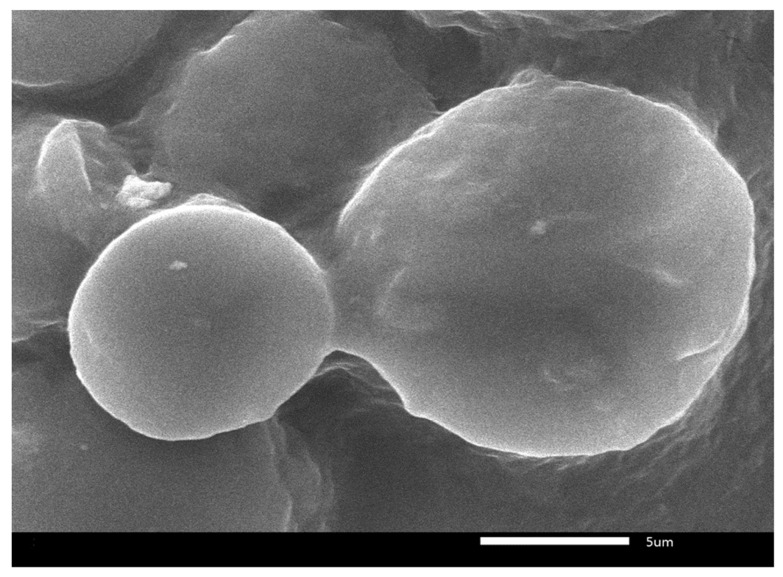
Surface SEM imaging film molecules 5000×; starch–glycerol–metal ion film.

**Figure 7:**
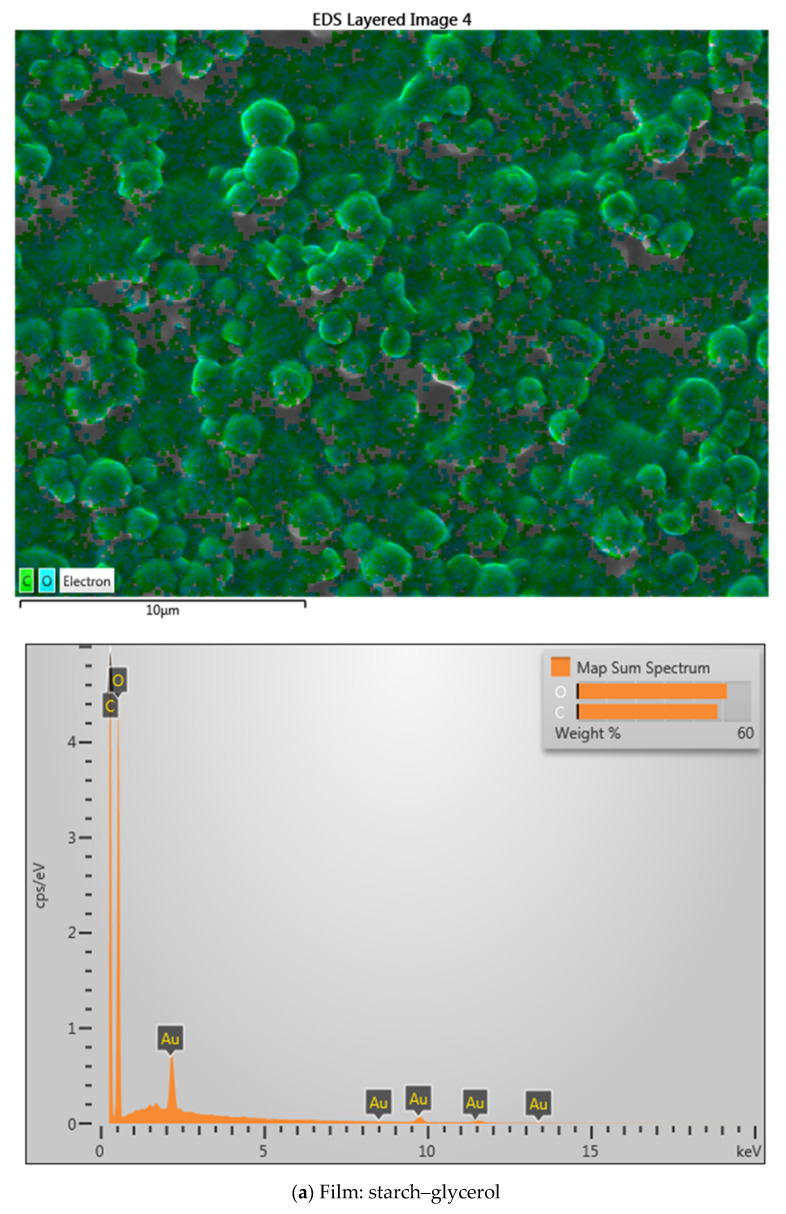

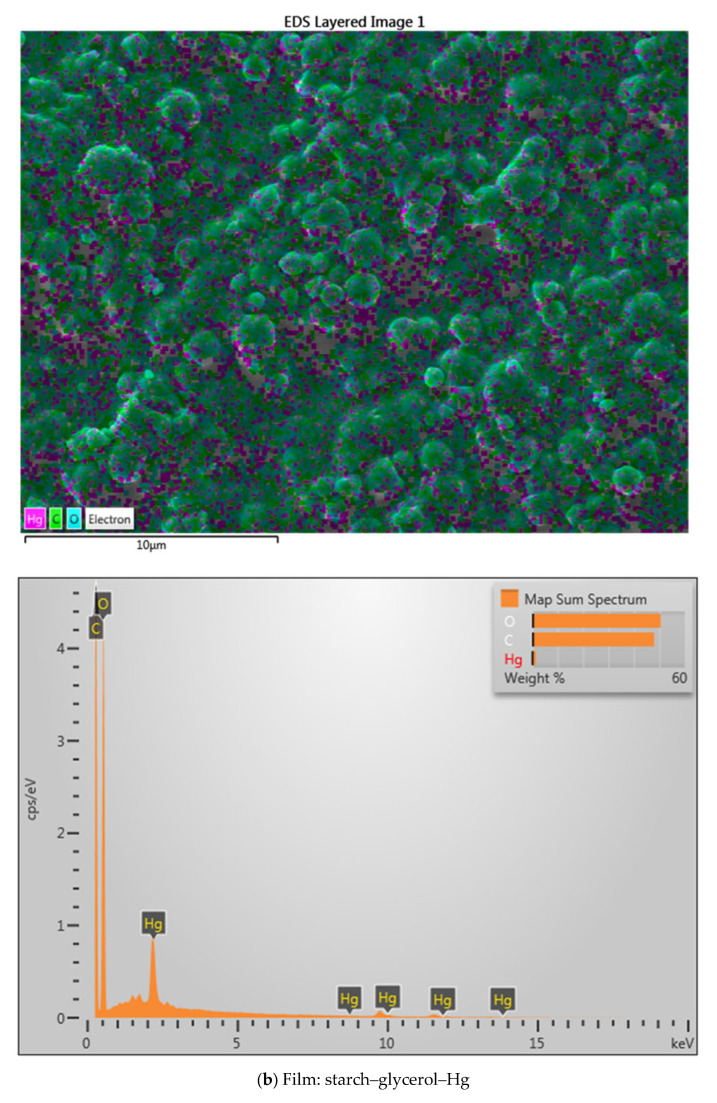

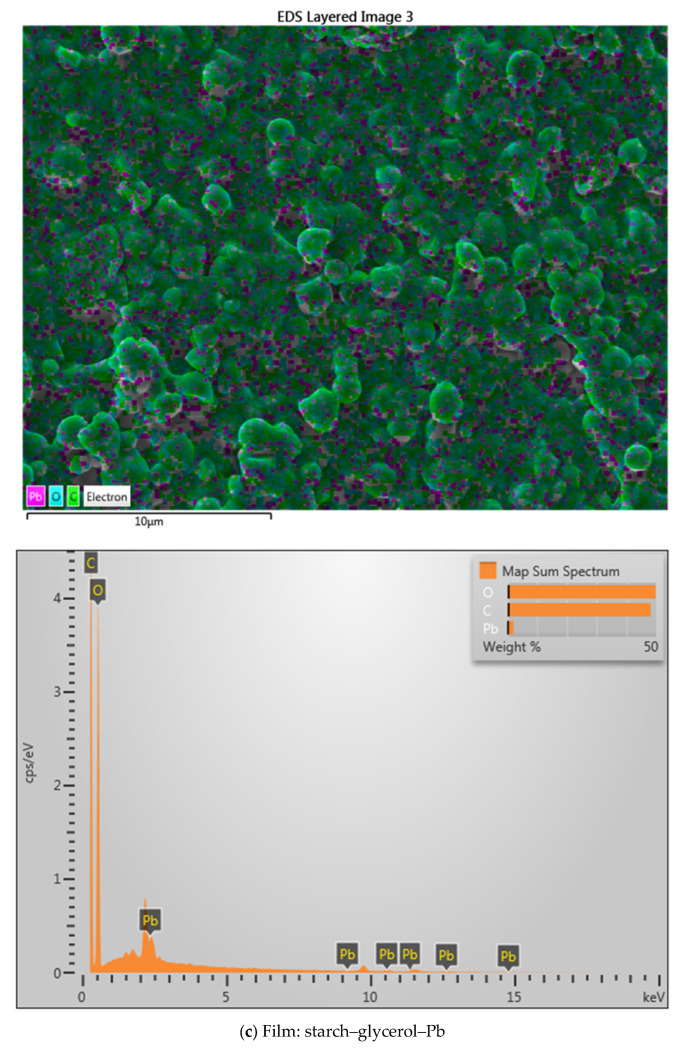

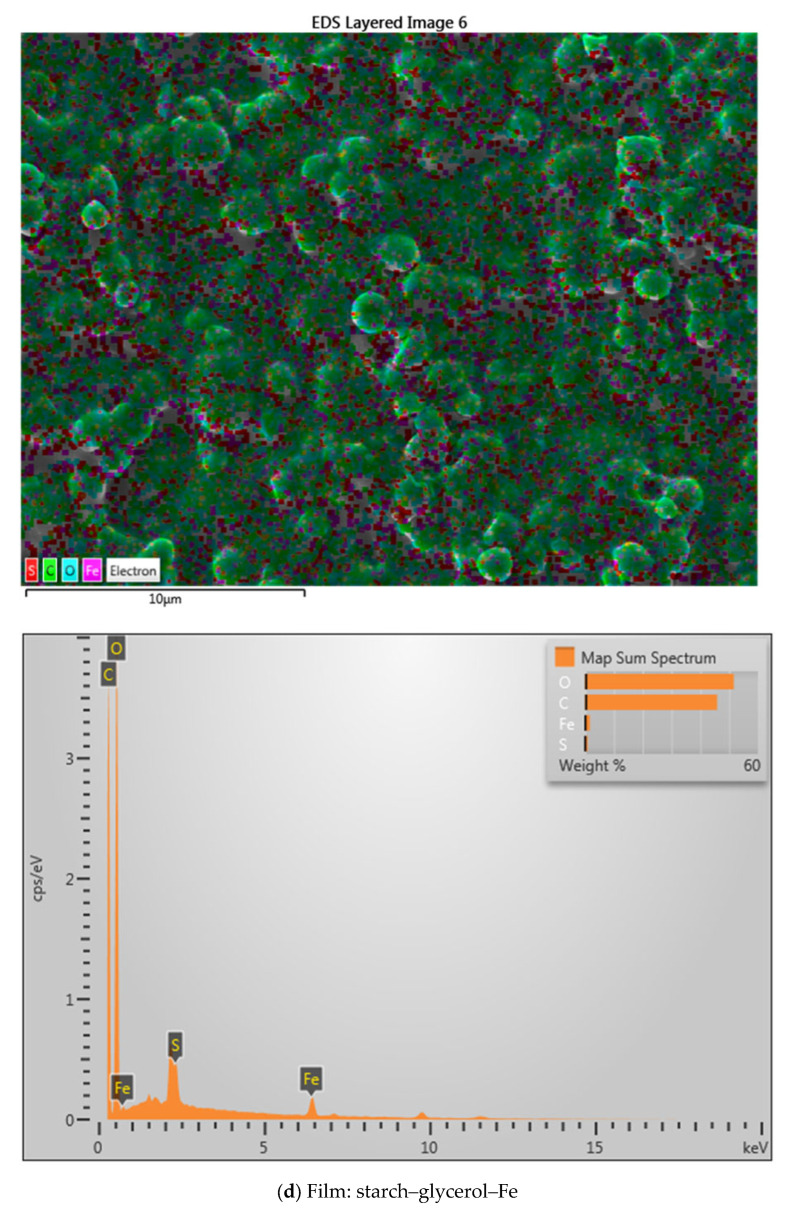

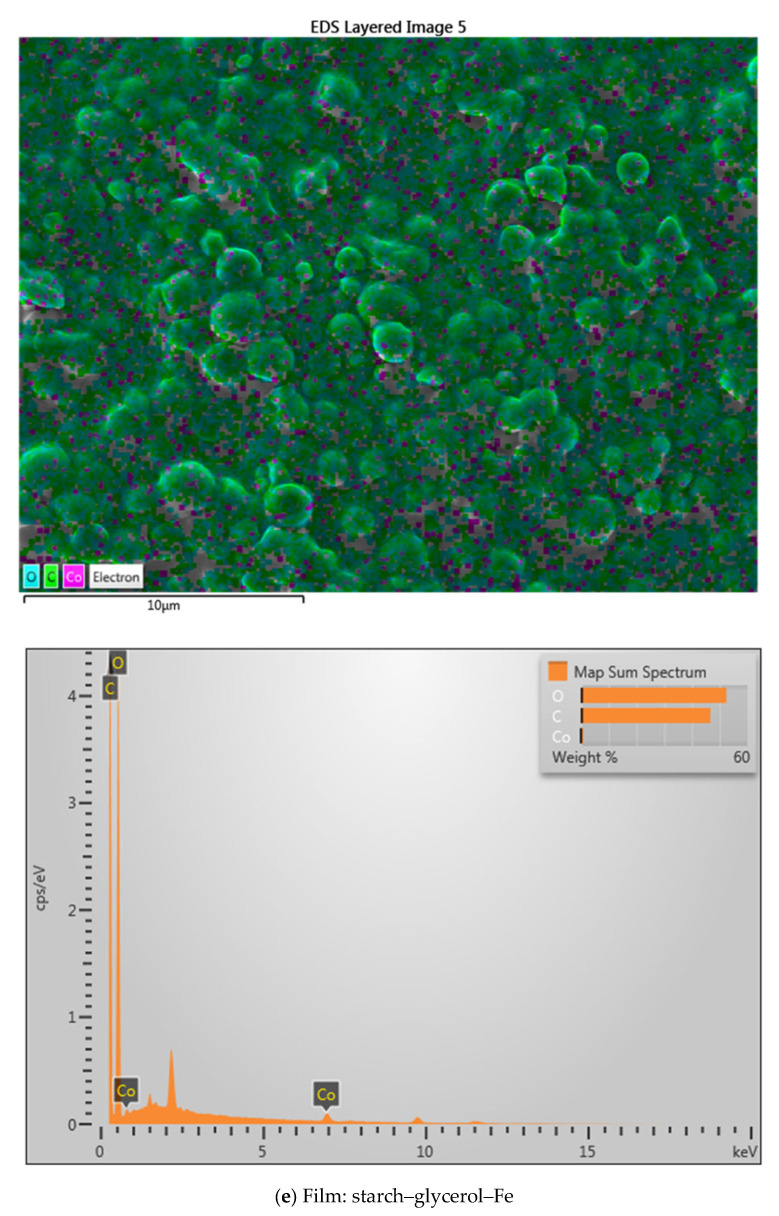
SEM characterization results of films magnified 500 times, and composition of material ion.

SEM analysis showed that metal ions adhered to the surface of the starch–glycerol gel molecules (Figure 5, Figure 6 and Figure 7). There is a difference between the surfaces of the starch–glycerol gel molecules and those of the starch–metal ion gel molecules. The difference is evident in the results of the SEM analysis at 5000× magnification (Figure 7). The surface appears smooth and flat in the starch–glycerol molecule. In contrast, the starch–glycerol–metal ion has a layer resembling a cloud or fog around the starch–glycerol molecule. Metal ions adhere to the surface of the starch–gelatin and form an amorphous structure.

Figure 5 is the result of taking pictures with a SEM tool. The image shows that the surface of the cassava starch–glycerol film has a dense shape and a flatter and neater surface.

Comparing Figure 5 and Figure 6, the cassava starch–glycerol molecules seem to have shadows and appear foggy. This can be the presence of metal ions attached to the film’s surface. SEM EDX testing was carried out to ensure the presence of metal ions.

The SEM EDX results in Figure 7 show metal attached to the film. The attached metal is relatively even; the added contaminant is a metal sample. The SEM results show that cassava starch–glycerol gel can interact with metal. An XRD test confirms the type of bond formed and identifies the surface area and pore size using BET analysis.

### 2.4. BET Analysis

Table 1 shows the results of the BET analysis of cassava starch powder samples and starch–glycerol films.

**Figure 8:**
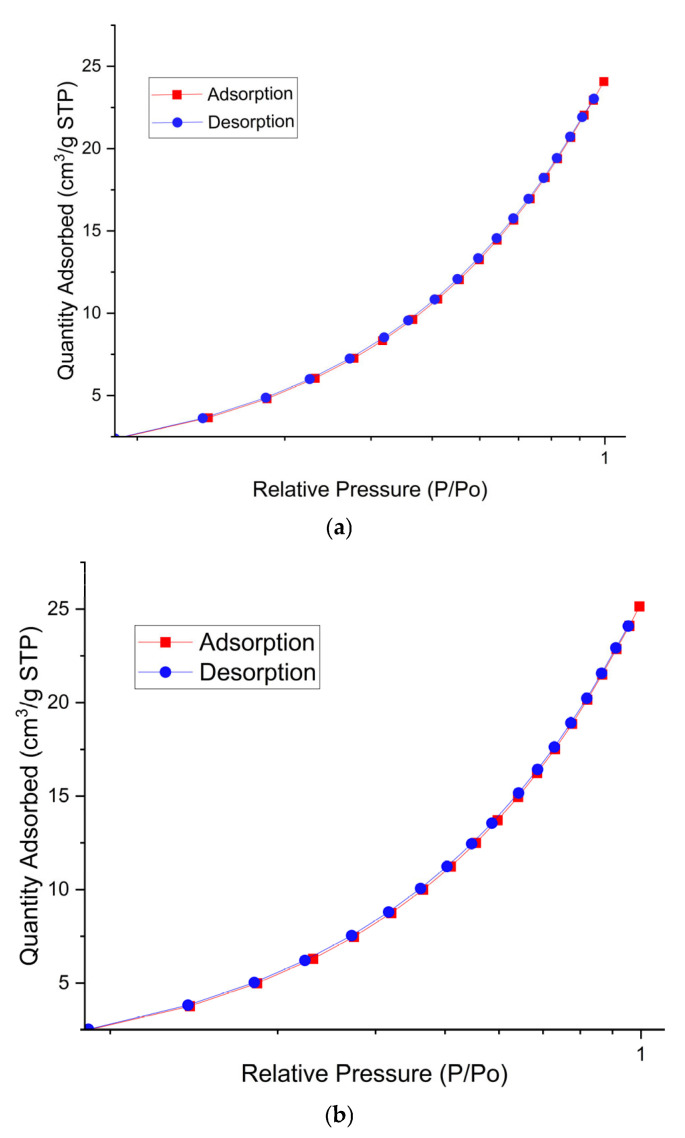
Graph of BET analysis: (**a**) cassava powder (raw material), and (**b**) starch–glycerol fixed film.

Based on the isotherm type, as shown in Figure 8, both samples are of the type 3 isotherm. Materials with type 3 isotherms have nonporous properties and energy adsorption [18]. Type 3 isotherms also exhibit complexities in physical adsorption [41,42]. It can be concluded that the starch–glycerol gel can absorb and bind a material.

### 2.5. XRF Analysis

Table 2 shows the results of XRF analysis. The samples analyzed by XRF were films formed by contact between the starch–glycerol gel and metal ions that had dried on glass, aluminum, and ceramic media.

While the media contaminated by metal ions showed relatively high concentrations, some decontamination gels showed higher values than the concentration of the solution dripped onto the media or material.

When stable metal ions, such as radionuclides, were prepared at a specific concentration to determine whether the starch–glycerol gel sufficiently adsorbs, they were produced at a particular concentration. However, in this study, the concentration of metal ions in the starch–glycerol–metal ion film was higher. The increased metal-ion concentration measured by XRF can occur because of water evaporation, which causes the level to rise above the initial concentration [38]. However, a decrease in concentration can occur due to the interaction or absorption of metal-ion solutions within the medium’s pores.

### 2.6. XRD Analysis

The XRD analysis showed that all starch–glycerol–metal film samples had an amorphous percentage above 65%, indicating that no chemical interactions can form crystals. The peaks observed in the starch–glycerol and starch–glycerol–metal ion films were at 2θ angles of 15°, 17°, 18°, 22°, and 23°, which is consistent with the research conducted by Luchese et al. (2017) [43].

**Figure 9:**
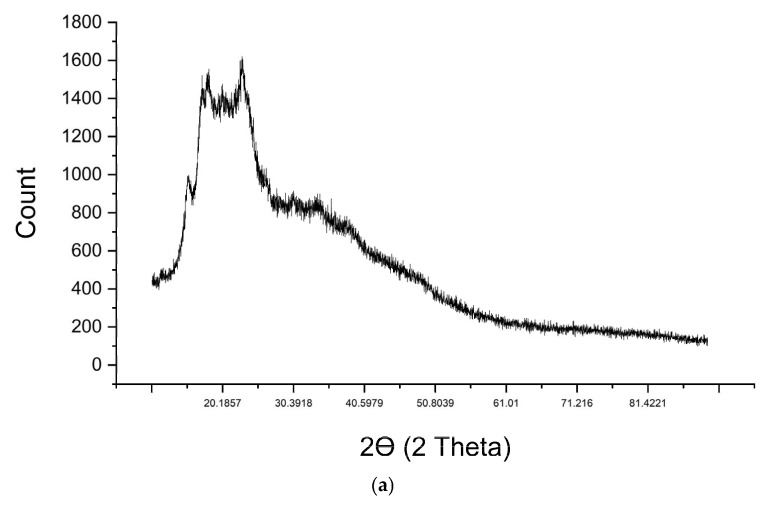

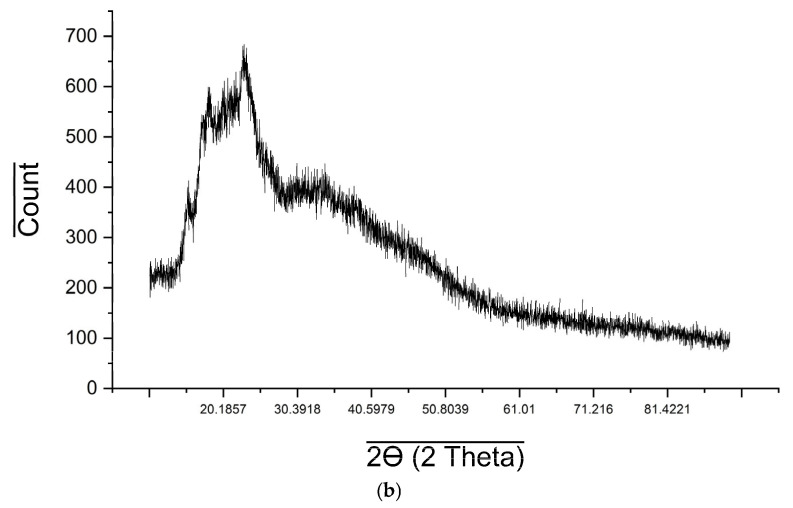

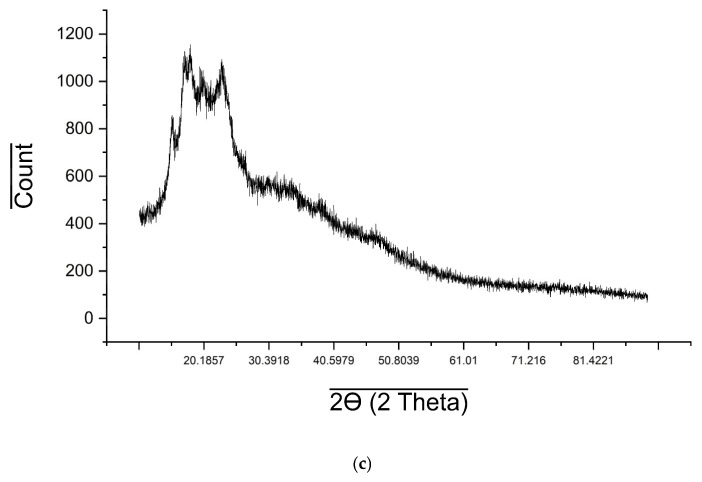

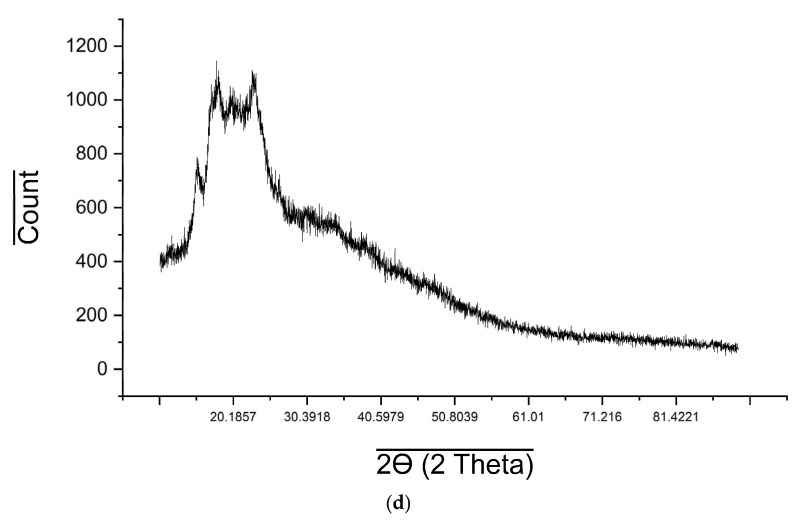

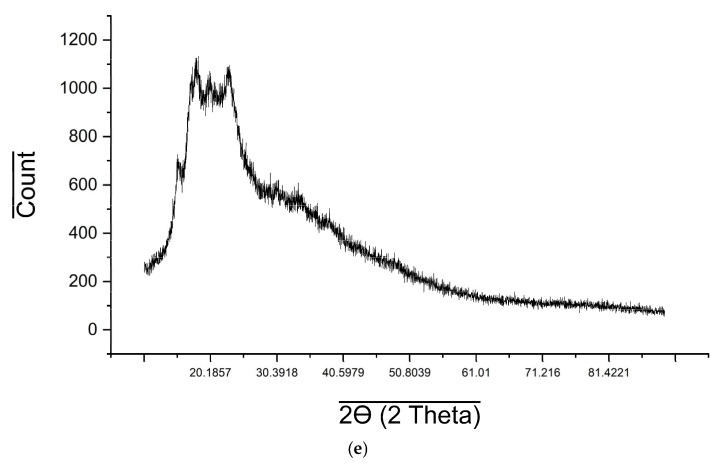
Characterization results for the XRD: (**a**) starch–glycerol, (**b**) starch–glycerol–Co, (**c**) starch–glycerol–Fe, (**d**) starch–glycerol–Hg, and (**e**) starch–glycerol–Pb.

Amorphous polymers exhibit irregular crystal structures. Amorphous polymers are flexible and transparent materials. Figure 9 shows amorphous at XRD analysis. The film resulting from the reaction between the starch–glycerol gel and metal ions is semi-crystalline and amorphous because it contains less than 35% crystals [44]. The crystallites in the film can originate from metal-ion salts that do not fully react, interact, or bond with the O-H groups originating from the polymer gel.

In addition, the starch–glycerol gel, after drying into a film, has a reasonably high surface area and small pore size, making it easier for it to interact with metal ions [33,45]. The reaction is as follows:

P-OH + M → P-OH-M

In the above,

P-OH = starch–glycerol gel polymer;

M = metal ion;

P-OH-M = starch–glycerol–metal ion film.

Ion bonding interactions can form through electrostatic interactions if there is contact between the gel and metal ions. The absorption or reaction can be improved by modifying the contact temperature. Additionally, several studies have shown that the contact time between the gel and metal ions is significantly influenced by the polymer structure of the gel material used [43]. Several studies on cassava starch-based hydrogels have shown that the absorption rate and the interaction of the gel with the metal ions are affected by the pH [46]. In further research, it will be necessary to add temperature variables for the incubation, the contact time, and the pH of the gel being tested, as well as the chelating materials or agents that increase the binding of metal ions. Therefore, the direct testing of materials contaminated with radioactive substances is required to ensure safety.

The high effectiveness of the decontamination gel in reducing surface contamination can make it an option for reactor decommissioning activities or radiation facilities that use open radiation sources. Until now, the polymers used for decontamination gel materials have been synthetic polymers such as PVA. Gels of PVA polymers and other mixed materials can reduce contamination levels and bind heavy-metal ions.

One example of the successful use of polymer gel composites for heavy-metal decontamination involved the manufacture of a gel coating composed of PVA combined with glycerol, EDTA, and bentonite [47]. The results of the SEM analysis showed that the decontamination film had heavy metals attached to the gel part [48]. Our research showed the same results: gels or films made from starch and glycerol can bind metals. This can be observed in Figure 7. The figure shows the presence of peaks from the standard metal ions, which are in accord with those of the contaminating metals used in this study.

Another study using a gel or polymer hydrogel as a decontamination gel material used PVA gel to decontaminate Mo-99 on glass, stainless steel, and wood surfaces [49,50]. The success rate of the decontamination ranged from 94% to 97% [51,52]. Research about gel decontamination showed that PVA gel combined with EDTA can reduce contamination by 53 to 98%, depending on the type of material surface [3]. It can be concluded that surface decontamination of materials using gel or hydrogel methods has great potential for reducing the level of contamination of heavy metals or radioactive substances.

In other studies, PVA and other materials, such as glycerol, are used to react with crosslinking to form polymer bonds that form a gel under normal conditions and will become a film when dry [49]. Therefore, this study used natural materials that are easy to obtain and inexpensive. Of course, this material was selected because it has properties or characteristics similar to those of PVA. Cassava starch is an agricultural commodity in Indonesia, and cassava starch can be processed into food. In Indonesia, cassava starch and technical glycerol are easy to acquire, and the price is more economical than PVA [25,29,30]. In this study, data were obtained indicating that cassava starch reacted with glycerol at a specific concentration; the material was then heated at a certain temperature to form crosslinks, which then became a gel and dried at a particular time. This behavior is similar to the properties of polyvinyl alcohol (PVA). The abundance of cassava starch and glycerol in Indonesia is undoubtedly very promising if cassava starch–glycerol gel is used for surface decontamination, especially for heavy-metal contamination, and this approach has potential for use in radioactive contamination in nuclear or radiation facilities.

One specification for radioactive decontamination is that the material does not react with radioactive materials, but can stick to the surface. Another classification is that the gel decontamination must be easy to apply [50]. Cassava starch–glycerol gel has met both criteria. This is proven by the results of the analysis carried out in this study. Cassava starch–glycerol gel is easy to make and easy to apply, and it can absorb metal ions on the surface of the material.

This can be an advantage because Indonesia has a TRIGA 2000 Reactor, which was the first research reactor owned by BATAN. This reactor was built in the Bandung Nuclear Area on 1 January 1964. It was inaugurated on 20 February 1965, with a capacity of 250 kW, under the name of the TRIGA Mark II Bandung Reactor. In 1971, the reactor capacity was increased to 1 MW, and in 1996, the capacity was increased again to 2 MW, at which point, the reactor was re-inaugurated on 24 June 2000 and renamed the TRIGA 2000 Bandung Reactor [53].

Having an age of more than 50 years, the TRIGA 2000 Bandung Reactor has previously experienced a suspension of its operating permit owing to the inappropriateness of the building structure and the potential for natural disasters due to earthquakes from the Lembang fault. These indications show that the TRIGA 2000 Bandung Reactor facility has undergone an aging process. In 2017, the TRIGA 2000 Bandung Reactor regained its operating permit, valid until 2027, with a maximum power limitation of 1000 kW. Given this, the National Research and Innovation Agency (BRIN) has a plan to carry out the decommissioning process at the TRIGA 2000 Bandung Reactor [54,55,56,57].

In the general document of the TRIGA 2000 reactor decommissioning plan, BRIN can include a decontamination method utilizing cassava starch–glycerin gel after conducting direct tests. By reducing the surface contamination associated with the decommissioning materials, the volume of waste can be reduced. Decreased waste volume will also reduce decommissioning costs, especially with respect to managing radioactive waste. In addition, it can reduce the area required for storage of the decommissioning waste. Reducing the level of contamination can also provide security and safety guarantees for radiation workers during the dismantling of nuclear reactors and their supporting facilities.

As to future work, we would need to research the application of the gel of starch cassava–glycerol directly to material contaminated with radioactive residues.

## 3. Materials and Methods

The cassava starch used is commercial starch sold in supermarkets under the brand name “Pak Tani Gunung” with a carbohydrate content of 87 g per 100 g of flour. Glycerol was used at concentrations of 80–95%. As for the molecular weight of the cassava starch components, amylose is a linear starch component with a molecular weight between 10⁵ and 10⁶ Daltons (g/mol) and amylopectin is a branched component of starch with a much higher molecular weight, ranging from 10^7^ to 10^9^ Daltons (g/mol). Overall, the average molecular weight (Mw) of the cassava starch ranges from 2.187 × 10^7^ g/mol to 5.7 × 10^7^ g/mol [58]. The purified or distilled water used had a conductivity of 0.055 µS/cm and a chemical oxygen demand (COD) content of 5 ppb.

The starch gel was prepared from packaged cassava starch flour (composition) 16% *w*/*v* (16 g), technical glycerol 10% *w*/*v* (10 g), and purified water added until the volume reached 100 mL. The solution was heated at 105–120 °C for 20–30 min to dissolve and mix it. The contaminated material was prepared by dripping a solution of stable metal ions that corresponded to the following radionuclides: Hg(II) 10,000 ppm, Pb(II) 5000 ppm, Fe(II) 10,000 ppm, and Co(II) 20,000 ppm. The solution was dripped on media in the forms of glass (object glass), aluminum metal sheets, and commercial ceramics used in buildings. The metal–ion solution drops were left at room temperature for 24 h. After the metal solution in the medium dried, a starch–glycerol gel was added to the contaminated area, and the sample was stored at room temperature for 24 h.

For identification and characterization of the starch gel, analysis was carried out using the following methods:

1. Fourier Transform Infrared Spectroscopy (FTIR): ATR Thermo Nicolet iS5. The spectra were read in the 400–4000 cm^−1^ frequency range in attenuated total reflectance (ATR) mode with a diamond crystal, 64 consecutive scans at a resolution of 4 cm^−1^.
2. Brunauer–Emmett–Teller (BET): Nova2000 Quantaqrom. Sample used without degassing. Outgas time, 3 h; outgas temp, 30 °C; analysis gas, nitrogen; bath temp, 77.3 K; press. tolerance, 0.100/0.100 (ads/des); equil time, 60/60 sec (ads/des); equil timeout, 240/240 sec (ads/des).
3. Scanning electron microscopy (SEM): Jeol. signal, secondary electron (SE); voltage, 20 kV; vacuum, high vacuum.
4. X-ray fluorescence (XRF): XRF Thermo Scientific Niton type XL3t 500 Analyzers. X-ray source, X-ray tube with gold anode (Au); maximum voltage, 50 kV; maximum current, 200 μA; maximum power, 4 watts; detector, high-performance semiconductor.
5. X-ray diffraction (XRD): Bruker D8. Scanning with Cu Kα radiation (λ = 1.5406 Å), operating at 40 kV and 40 mA. The scanning range of 2θ was from 5 to 90°.

## 4. Conclusions

This study found that cassava starch combined with glycerol at 16% *w*/*v* cassava and 10% *w*/*v* glycerol can form a gel-shaped polymer that adsorbs stable metal ions corresponding to radionuclides on glass media, aluminum metal plates, and ceramics. The starch–glycerol gel forms a peel-off film that is easily peeled off when dry. This suggests that the starch–glycerol gel can be an alternative method used to decontaminate material surfaces. The adsorption capacity of this starch–glycerol gel is supported by the many O-H groups in the polymer, which can bind metal ions through electrostatic interactions. Another factor contributing to the good adsorption capacity of the starch–glycerol gel is that after the gel transforms into a film, its surface area value is relatively high, and the pore size is small. This is similar to the properties of polyvinyl alcohol (PVA). The abundance of cassava starch and glycerol in Indonesia is undoubtedly very promising if cassava starch–glycerol gel is used for surface decontamination, especially if used for heavy-metal contamination and potential radioactive decontamination in nuclear or radiation facilities.

## Figures and Tables

**Table 1:** BET analysis results.

Material Name	Surface Area (m^2^/gram)	Mean Pore Diameter (nm)	Total Pore Volume (cm^3^/gram)
Cassava powder (raw material)	1202.047	0.1774	1.064
Starch–glycerol fixed film	33,703.643	0.1776	2.993

**Table 2:** Elemental analysis of metal on gel.

No.	Metal Ions	Concentration (ppm)	Values Read on Resulting Film—Decontamination of Media		
			Glass	Ceramics	Aluminum
1	Aquadest	0	0	0	0
2	Hg	10.000	6.600	19.957	4.309
3	Pb	5.000	18.362	6.081	8.552
4	Fe	10.000	68.419	123.001	111.970
5	Co	20.000	28.761	16.222	64.587

## Data Availability

The original contributions presented in this study are included in the article. Further inquiries can be directed to the corresponding authors.
